# Tuning of the magnetic properties of iron oxide core–shell nanoparticles by controlled oxidation

**DOI:** 10.3762/bjnano.17.93

**Published:** 2026-10-06

**Authors:** Aladin Ullrich, Michael Kühn, Juliane Ohmer, Manfred Albrecht

**Affiliations:** 1 Institute of Physics, University of Augsburg, Universitätsstraße 1, 86159 Augsburg, Germany

**Keywords:** core–shell, exchange bias, iron oxide, memory effect, nanoparticles, superparamagnetic blocking temperature

## Abstract

Superparamagnetic iron oxide core–shell particles with average sizes of 10 and 14 nm were synthesized by thermal decomposition of an iron oleate precursor in a high-boiling solvent. Sodium oleate was used as surfactant to produce cubic particles. The particles show a wustite (FeO) structure in the core and spinel structures of maghemite (γ-Fe_2_O_3_) or magnetite (Fe_3_O_4_) in the shell. Due to interfacial coupling of the antiferromagnetic core to the ferrimagnetic shell, the particles exhibit a pronounced exchange bias (EB) effect, resulting in a shifted hysteresis loop when measured below the superparamagnetic blocking temperature. The EB behavior was systematically investigated depending on the core/shell ratio, which was achieved by artificial oxidization of the particles. With decreasing core/shell ratio, the superparamagnetic blocking temperature, the coercivity, as well as the EB field decrease. In addition, magnetic memory measurements indicate the presence of a superspin glass state at low temperatures.

## Introduction

Magnetic nanoparticles from transition metal oxides are a subject of intense research due to their high potential in various applications. In particular, iron oxide nanoparticles can be used in medicine for diagnostics [1] and therapeutics [2–4], in spintronic [5] and sensor devices [6], as well as in environmental applications like water treatment [7–9] or catalysis [10]. Since the physical properties of nanoparticles rather depend on their size, size distribution, and shape, it is important for most applications to have monodispersed particles, that is, particles with a sharp size distribution and uniform shape. A common way to produce such monodispersed particles is the thermal decomposition of organo-metallic precursors, such as oleates in high-boiling solvents under an inert atmosphere [11–12]. The particles prepared by this method often show a core–shell structure due to reduction and oxidation processes during the preparation [13–15]. Iron oxides can exhibit antiferromagentic (AFM) as well as ferro- (FM) or ferrimagentic (FiM) order [16]. The magnetic properties of such particles strongly depend on their internal structure with interfaces between different iron oxide phases. For instance, a FM/FiM structure in the vicinity of an AFM phase can lead to magnetic exchange bias (EB) effects [17–18]. This effect is typically observed in systems where the Curie temperature of the FM or FiM is higher than the Néel temperature (*T*_Neel_) of the AFM when the system is cooled below *T*_Neel_ in the presence of an external magnetic field. This process leads to a shift of the magnetic hysteresis loop. Additionally, the phenomenon of superparamagnetism can be present as well [19]. This is the case when the small particles cannot keep a magnetic orientation due to thermal fluctuations of the magnetization above a critical temperature called superparamagnetic blocking temperature *T*_B_. Besides the size of the particles, the magnetic anisotropy of the system, which is (among others) influenced by the EB effect has an impact on *T*_B_ [20]. In addition, the coercivity is expected to depend on the crystallite size [21].

In this study, superparamagnetic iron oxide core–shell nanoparticles were synthesized, and their structural and magnetic properties were investigated. Due to interfacial coupling of the AFM wustite core with the FiM γ-Fe_2_O_3_/Fe_3_O_4_ shell, the particles exhibit an EB effect, which can be controlled by changing systematically the core/shell ratio. In addition, due to this strong intraparticle interaction, the presence of a superspin glass state was analyzed by memory experiments.

## Experimental

Iron oxide nanoparticles were synthesized by thermal decomposition of an iron(III) oleate complex precursor in 1-octadecene, as first described by Hyeon and coworkers [12,22]. The precursor was synthesized shortly before the particle synthesis to avoid ageing effects [23]. Typically, for the precursor synthesis, 0.67 g of sodium hydroxide are dissolved in 8 mL of distilled water in a three-necked round bottom flask, equipped with a reflux condenser, nitrogen inlet and thermometer. Under permanent stirring, 1.41 g of FeCl_3_·6H_2_O are added to this solution. Afterwards, 18 mL of *n*-hexane as well as 10 mL of ethanol are added. Finally, 4.97 g of oleic acid is added, and the reaction mixture is heated up to its reflux temperature (≈68 °C) for about 4 h under nitrogen atmosphere to produce the iron oleate precursor. After the synthesis, the precursor was washed three times with 10 mL of H_2_O in a separation funnel, followed by a drying step under vacuum at ≈50 °C for 4 h to remove excess solvents. The product was a dark-brown and highly viscous material. For the basic particle synthesis, 1 g of the precursor was loaded into 10 mL of previously degassed 1-octadecene. For shape control, 0.34 g of sodium oleate as surfactant was added [24]. The reaction solution was heated up to its boiling point of 318 °C for 30 min under permanent stirring, then the solution was cooled down to room temperature. For all transfers, a Schlenk line was used to avoid oxygen contact. All experiments were conducted under nitrogen atmosphere. A constant nitrogen flow of about 1 mL/s was applied during all experiments. The applied oxidation process to change the core/shell ratio was performed in the same three-neck flask. For this purpose, the solution was heated up again, but this time 1.5% of oxygen was added to the nitrogen atmosphere. The experiment was conducted under reflux setup. However, it was observed that the oxidation completes after about 20 min during the heat-up process before a significant reflux started. Two sample runs were performed: (i) at low concentration (2 mL of the as-prepared particle solution dissolved in 10 mL of 1-octadecene, 1.5% oxygen atmosphere) to evaluate the oxidation process by (scanning) transmission electron microscopy ((S)TEM) and X-ray diffraction (XRD). Only the initial and final (fully oxidized) sample was taken for XRD analysis due to the high amount of material required for this kind of analysis; and (ii) at high concentration (full amount of as-prepared solution with additional 15 mL of 1-octadecene) for magnetic characterization and TEM analysis (1.5% oxygen, seven samples of 2 mL taken between 60 and 244 °C). After taking the samples, they were diluted with *n*-hexane, and the particles were precipitated with isopropanol and centrifuged to separate them. This cleaning procedure was applied three times. Finally, the particles were dispersed in *n*-hexane and stored until investigation or further preparation under ambient conditions. For the investigation by TEM, a carbon-coated copper grid for TEM analysis was dipped into the particle–hexane dispersion for about 1 s and dried under ambient conditions. All TEM images were taken without further treatment. For the electron energy loss spectroscopy (EELS) measurements, the samples were cleaned ≈1 min in air plasma to remove organic residues that cause strong carbon contamination on the sample during the scanning TEM investigation with a focussed beam. According to TEM images before and after plasma treatment, the core/shell ratio was not affected by this treatment; also, no sample degradation was observed during (S)TEM analysis. For XRD investigation, the particles were dripped on a glass substrate and dried under ambient conditions. Several droplets were applied until a closed layer of particles was formed. For superconducting quantum interference device (SQUID) magnetometry investigations, typically 5–10 mg of the particles were carefully dispersed in a resin (Ebalta AH 140) by a MICCRA D1 Pico dispenser. The resin is used to avoid particle movement during the measurement as well as to minimize cluster formation. These resin samples were directly prepared in capsules used for magnetic measurements. By the assumption of a perfect dispersion without clusters and taking into account the sample mass and size, and the individual particle size, the distance between the particles is about 200–300 nm and hence, particle–particle interaction is minimized. This has been confirmed by scanning electron microscopy on a cut resin piece. The used TEM was a JEOL NEOARM F200 instrument, operated at 200 keV beam energy, equipped with a GATAN Continuum S Energy loss spectrometer. The EELS measurements were performed in DualEELS^TM^ mode, that is, the zero-loss region was measured together with the high-loss region. The zero position of all spectra was recalibrated in a post-processing step by using the zero-loss peak to get rid of slight shifts of the energy scale by external influences or system instabilities. The used magnetometer was a Quantum design MPMS3 system.

## Results and Discussion

### Structural properties

**Figure 1:**
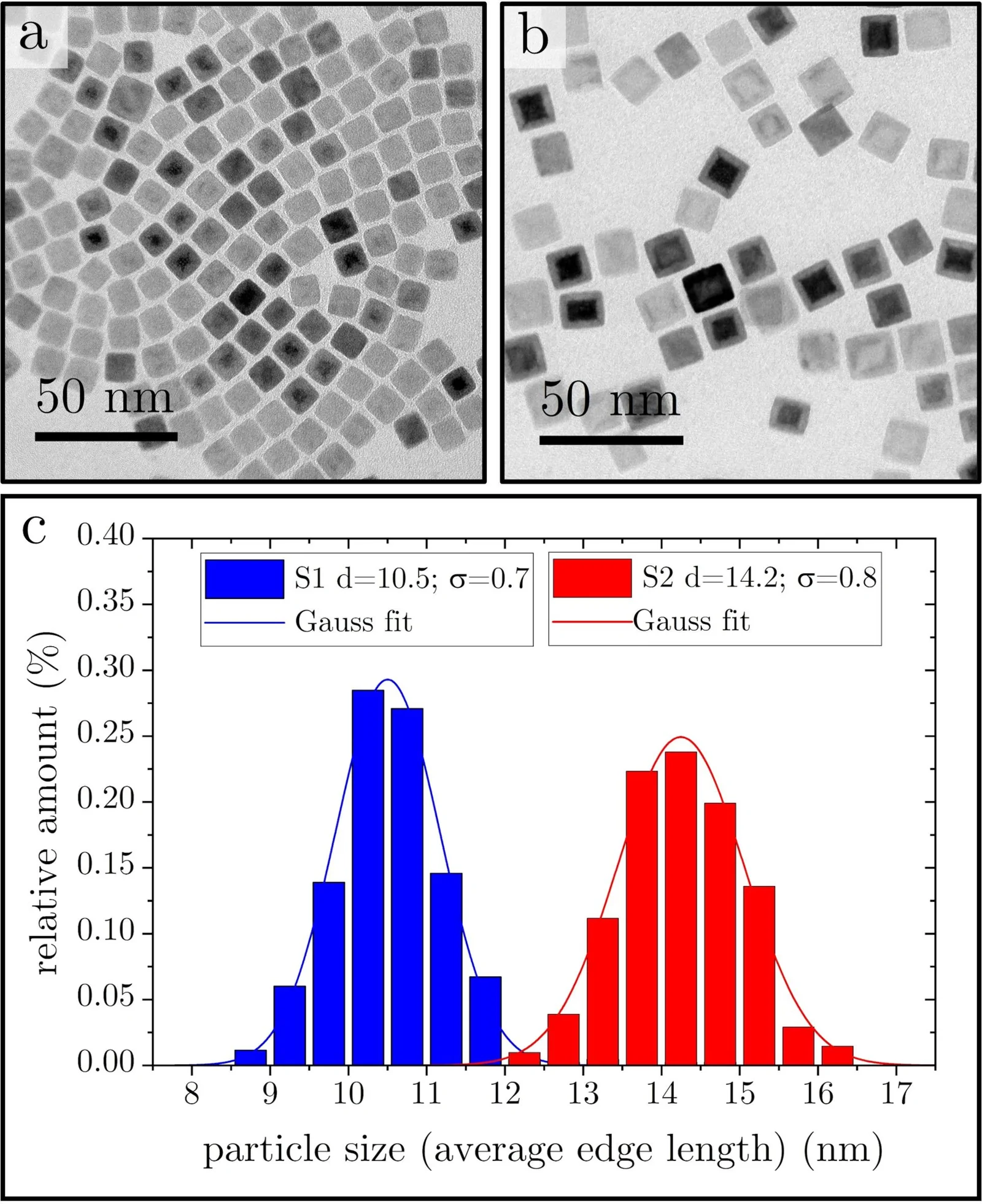
TEM images of two different samples with size distribution plots. (a) Sample S1 with particles exhibiting an edge length of about 10 nm; (b) sample S2 with particles exhibiting an edge length of about 14 nm; (c) histograms with Gaussian fits for size evaluation.

Shape, size, and structure of the produced particles were analyzed by (S)TEM. The particles are composed of a core and a shell, as can be seen in Figure 1a and Figure 1b for two different samples (S1 and S2, respectively) with slightly different particle sizes because of different aging times of the precursor [23] and slightly different heating rates [25]. Figure 1c shows the corresponding size distribution of the samples with diameters of *d*_S1_ = 10.5 ± 0.6 nm and *d*_S2_ = 14.2 ± 0.7 nm. For the histogram, more than 200 particles were measured from each sample. The core consists of AFM wustite (FeO), while the shell appeared as one of the spinel structures of FiM maghemite (γ-Fe_2_O_3_) or magnetite (Fe_3_O_4_). These findings are consistent with earlier studies [14,26]. It is known [13,22] that during the decomposition of the precursor, Fe^3+^ is reduced to Fe^2+^ because of reducing side products like H_2_ or CO. Afterwards, the particle surface oxidizes during the cleaning process under ambient conditions [26], resulting in a stable core–shell structure. A recent report by Lohr et al. [27] states that the core–shell structure is only obtained with an intermediate step during the synthesis, while the same protocol without this step leads to smaller particles without core–shell structure. In our synthesis protocol, no such step is necessary to obtain core–shell particles, which is in agreement with earlier reports [13–14,25].

**Figure 2:**
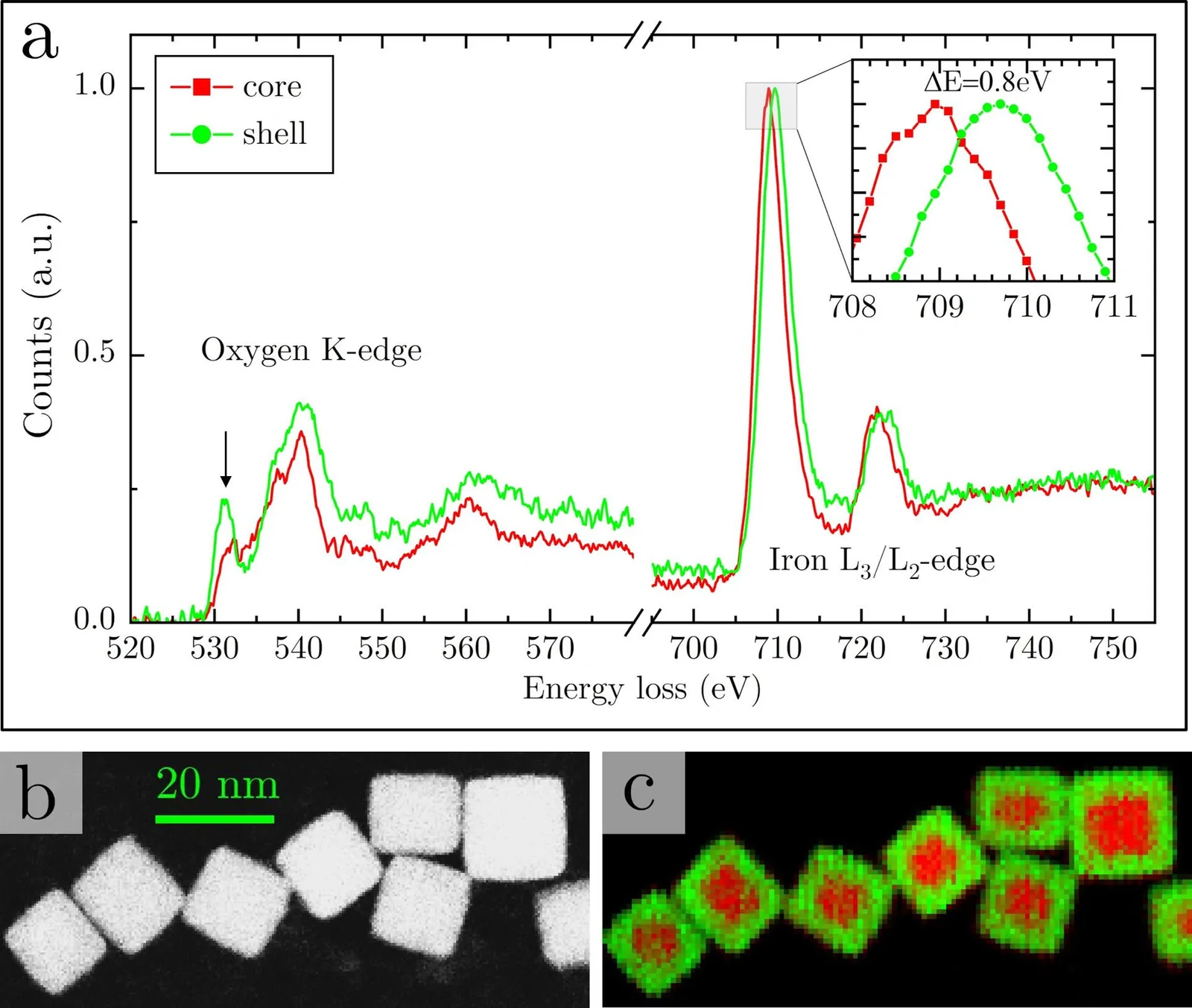
EELS analysis of the core–shell structure. (a) EEL spectra of the core and shell region. The pre-peak at the oxygen K-edge is marked with an arrow. Inset: magnified region of Fe L_3_ peak position. All spectra were recorded in Dual EELS^TM^ mode (see Experimental part for details) with a dispersion of 0.15 eV/channel. (b) STEM dark-field image. (c) Color coded chemical map representing the chemical shift according to (a). Red areas represent the lower oxidation state (Fe^2+^), while green color represents the shift of the L_3_ edge due to the higher oxidation state of Fe^3+^.

To prove the composition, electron energy loss spectroscopy (EELS) was performed. A typical EELS analysis of a core–shell particle is shown in Figure 2a. There are two features that are of interest: First, as shown in the inset, the position of the Fe L_3_ edge varies from the core to the shell, indicating a different oxidation state [28]. Second, there is an intense pre-peak at the oxygen K edge in the shell spectrum. This is an indicator for the chemical environment and only present in the spinel structures of magnetite and maghemite. The wustite structure does not show this feature [29]; hence, in the core region this pre-peak is weaker. But as the core is surrounded by the shell at the bottom and top of the particles, it does not vanish completely in the measurement. Figure 2b shows a dark-field image of several particles. At every pixel of Figure 2b, an EEL spectrum was taken. In Figure 2c, a color-coded chemical map is shown, created from fitting reference spectra characteristic for the core and the shell region to the measured spectra by linear combination. The fitting coefficient is represented by the green (for the shell) and red (for the core) color and visualizes the shift of the Fe L_3_ peak due to the different oxidation state of the iron atoms.

**Figure 3:**
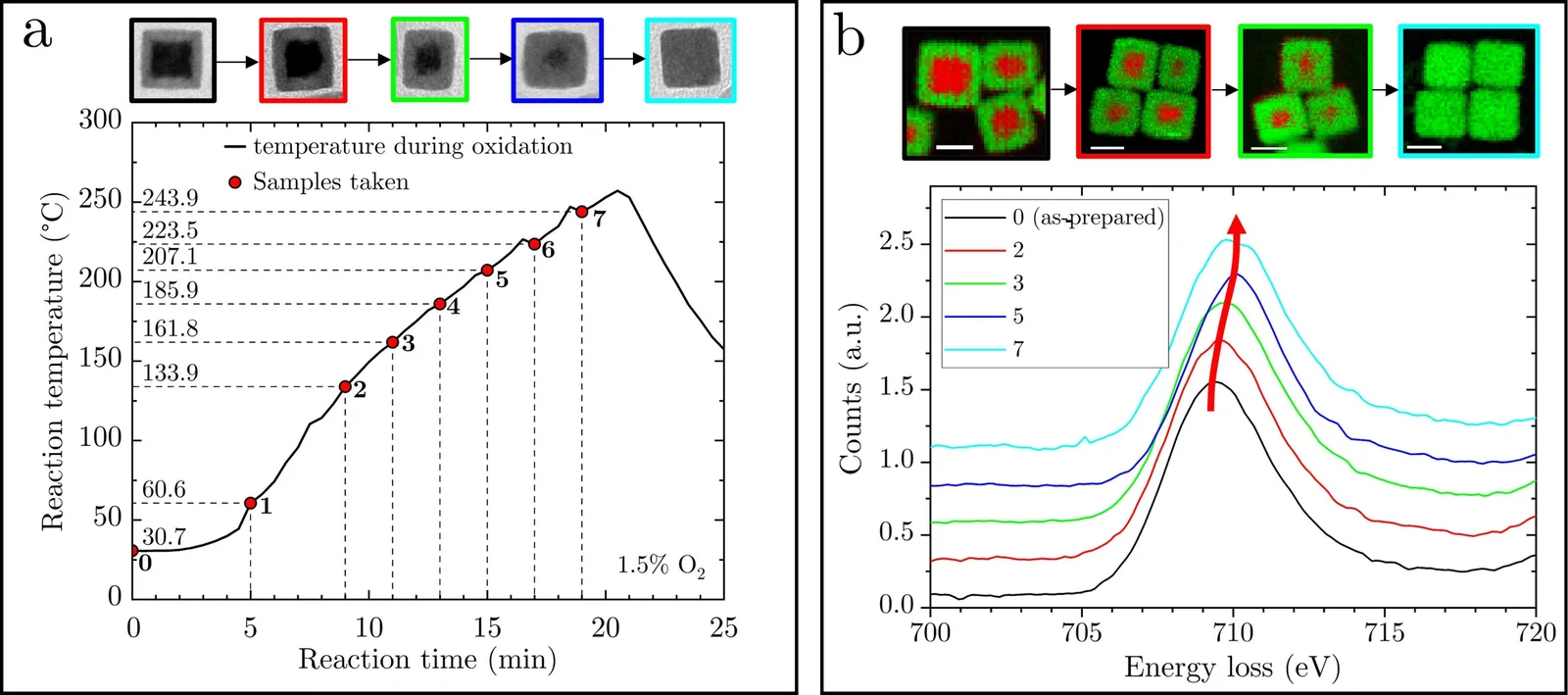
Artificial oxidation process. (a) Time and temperature treatment of the samples during the artificial oxidation process. The small dips in temperature arise because of short openings of the flask to take the samples. Top, from left to right: TEM images of particles of samples S2_0_*_,_*_2_*_,_*_3_*_,_*_5_*_,_*_7_ with different core/shell ratios. (b) EELS Fe L_3_ edge analysis of the samples, averaged over several 100 particles. The Fe L_3_ edge shift indicates the oxidation process. Top: chemical map of sample S2_0_*_,_*_2_*_,_*_3_*_,_*_7_ to visualize the shrinking of the core. Scale bar: 10 nm.

Sample S2 was then oxidized in a controlled way by heating up again in an atmosphere with 1.5% oxygen. By doing so, the core/shell ratio could be successfully tuned until the core had completely vanished. During the oxidation process, in total seven samples were taken, labeled S2*_N_* (with *N* = 0–7), as summarized in Figure 3a. The leftover of S2 after cooling down the reaction mixture gave one more sample denoted as sample S2_8_. Figure 3b shows the Fe L_3_ edge of the EEL spectra recorded over several hundred particles for samples S2_0_*_,_*_2_*_,_*_3_*_,_*_5_*_,_*_7_. There is a clear shift of the peak to higher energies, indicating the continuous oxidation of iron in the particles with oxidation time. TEM and chemical maps are shown on top and as inset, proving the growth of the shell and the steady disappearance of the core.

**Figure 4:**
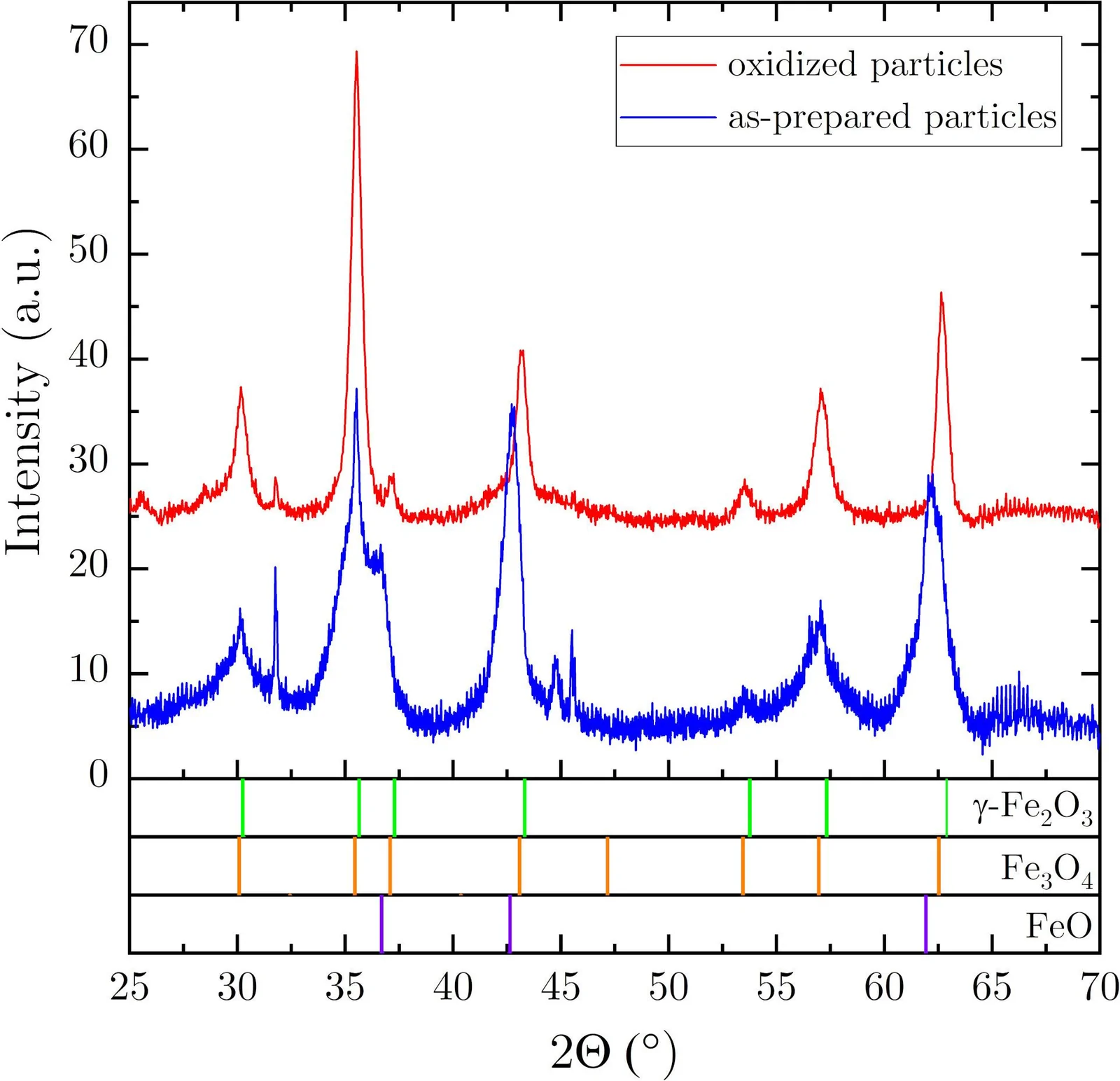
X-ray Θ–2Θ measurements of an as-prepared sample and an oxidized sample (prepared parallel to S2). Green, orange, and violet markers mark the peak positions of maghemite, magnetite, and wustite, respectively [16,26].

To confirm the change of the phases inside the particles during the oxidation process, X-ray diffraction was performed before and after the oxidation. Typical Θ–2Θ scans are shown in Figure 4 for a sample prepared parallel and with same parameters to S2 (due to the high amount of material required for XRD, it was not possible to take more samples and to investigate the same sample by TEM, SQUID, and XRD). It is clearly visible that the as-prepared sample (before oxidation) contains both wustite and one of the spinel phases (magnetite or maghemite), with quite broad peaks due to the small coherence volume of the core and shell. After the oxidation process, the wustite content is almost no longer detectable, while the peaks of the spinel phase get sharper due to the larger coherence volume. The marked peak positions are calculated from the structural data found in the literature for maghemite, magnetite [16], and wustite [26]. It was not possible to assign the spinel phase clearly to the maghemite or the magnetite structure because of their similarity and the nanocrystalline nature of the sample.

### Magnetic properties

#### Superparamagnetism and exchange bias effect

**Figure 5:**
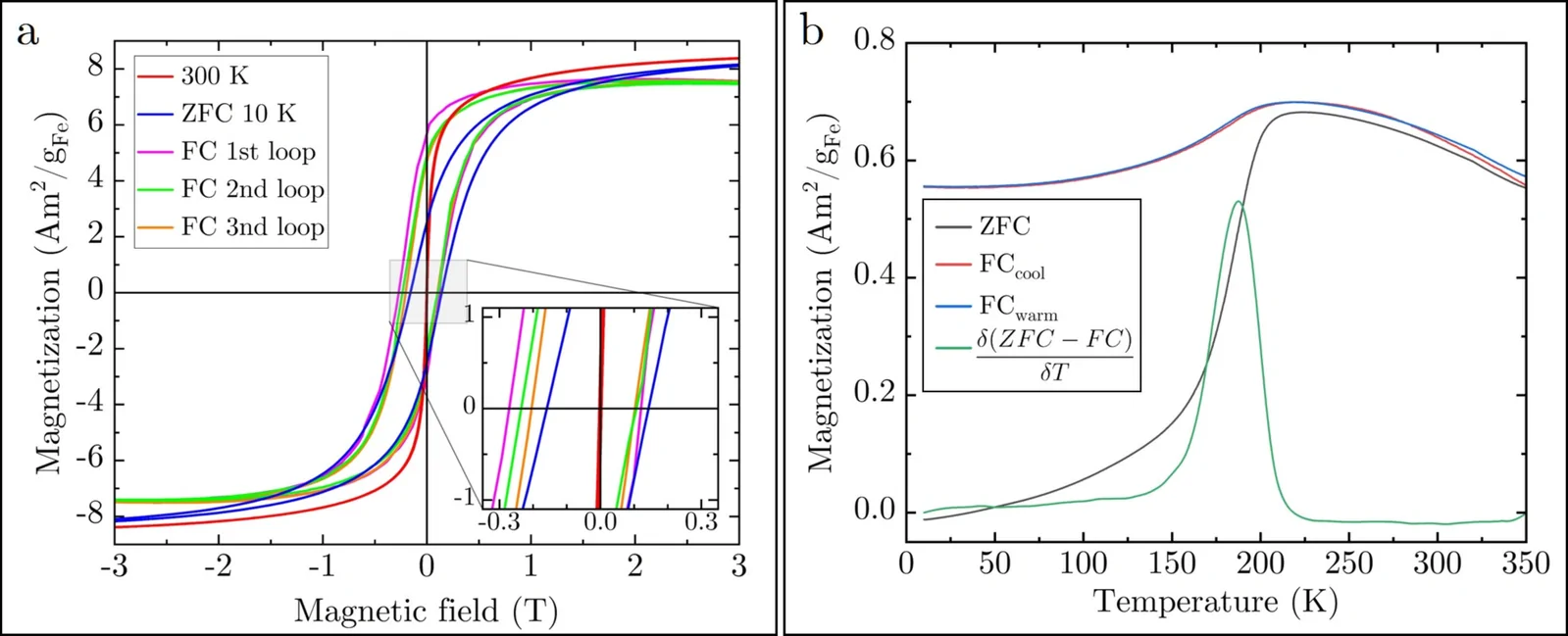
(a) RT, ZFC, and FC hysteresis curves of sample S2_0_. Note the training effect in the FC measurement. (b) ZFC and FC magnetization measurement of sample S2_0_ (with a guiding field of 50 Oe) with *T*_B_ = 190 K determined by the derivative-of-differences method [30–32].

Since the wustite core is antiferromagnetic and the spinel shell is ferrimagnetic [16], the magnetic properties are expected to change with changing the core/shell ratio by the controlled oxidation. In the following, the dependency of the blocking behavior, the coercivity, the exchange bias, and memory effects on the volumetric core/shell ratio are shown. The core/shell ratio was determined from TEM images as well as EELS mappings. In Figure 5a, magnetic *M*–*H* hysteresis loops measured by a superconducting quantum interference device-vibrating sample magnetometer (SQUID-VSM) at 300 and 10 K of sample S2_0_ are presented. The loop at 300 K shows no hysteresis, while the loop taken at 10 K reveals a significant coercivity (*H*_C_) of about ±148 mT and remanence (2.5 Am^2^/g_Fe_). This behavior is characteristic for superparamagnetism, which is further probed by field-cooled (FC) and zero-field-cooled (ZFC) magnetization curves, as presented in Figure 5b. For the FC case, the warming and cooling curves are shown, which do not reveal a significant difference. The data were recorded with a temperature rate of 5 K/min and a measuring guiding field of 50 Oe. During the field cooling, a field of 70 kOe was applied. The ZFC curve shown in Figure 5b was acquired by first cooling the sample in zero field from 300 down to 10 K and then switching on the guiding field of 50 Oe and measure the magnetization during warming. The magnetic blocking temperature (*T*_B_) was determined from the maximum of the derivative of the difference of the ZFC and FC_warm_ curve 
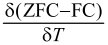
 according to literature [30–32], which gives in this case a value of about 190 K for sample S2_0_.

**Figure 6:**
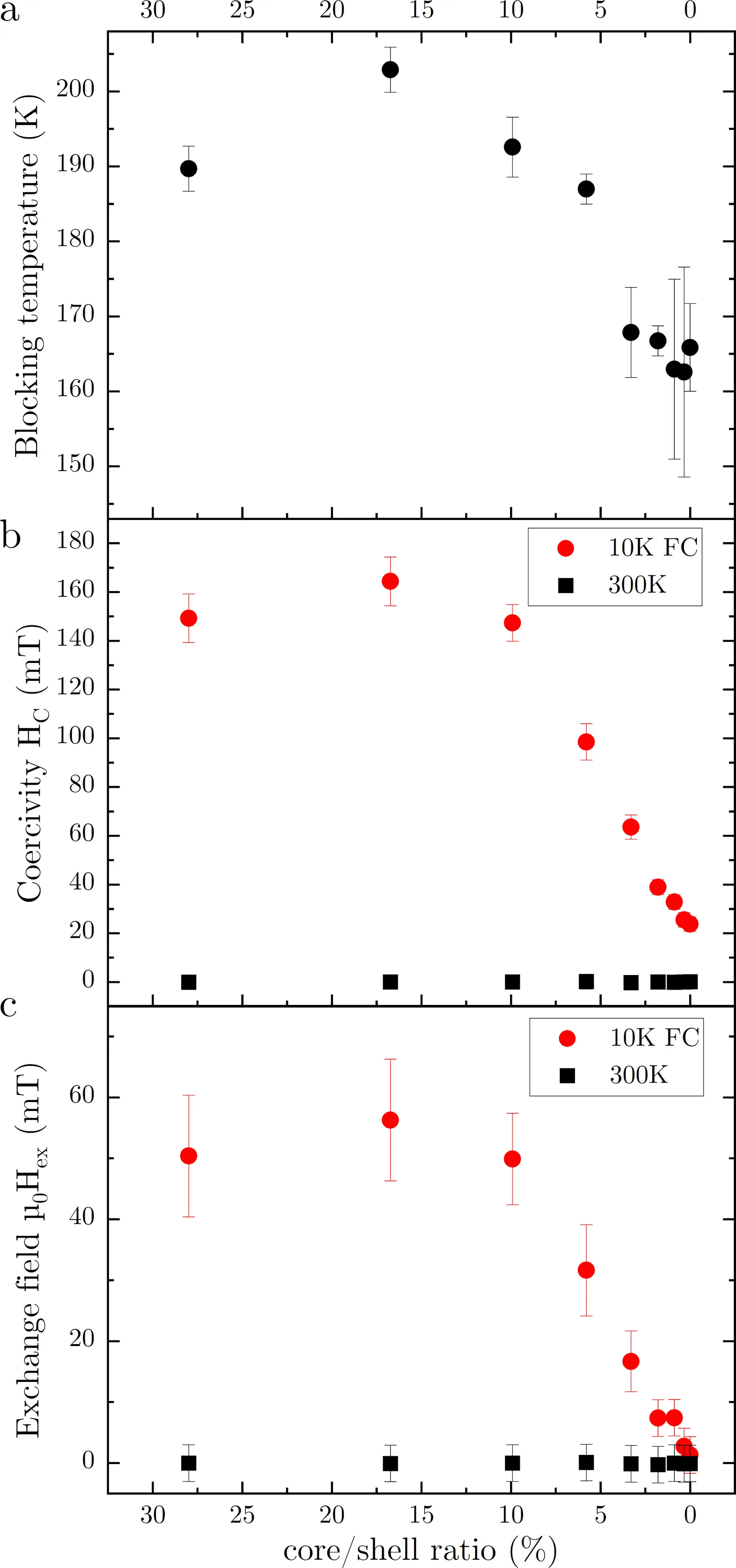
(a) Superparamagnetic blocking temperature, (b) coercivity, and (c) EB field as functions of core/shell ratio (samples S2_0_ to S2_8_).

Figure 6a summarizes the blocking temperatures as function of the core/shell ratio (samples S2_0−8_). As the volume of the ferrimagnetic spinel phase is increasing with oxidation, an increase in blocking temperature is expected. However, this is only initially observed followed by a decreasing *T*_B_, as will be discussed later.

Due to interparticle interfacial coupling of the core–shell structure, also an EB effect is expected. After field cooling in 70 kOe down to 10 K, the *M*–*H* loop of sample S2_0_ shows a shift of about 75 mT, which decreases to about 50 mT after the third loop cycle, as displayed in Figure 5a. Because of this known training effect [18], all samples were trained three times for the measurement of the EB field. The EB fields were calculated by (*H*_C+_ − *H*_C−_)/2, with *H*_C_*_+/−_* being the coercivity values on the positive and negative side of the loop, respectively. The EB field and the coercivity measured from the *M*–*H* hysteresis loops at 10 K after FC (in the blocked state) are shown in Figure 6b and Figure 6c, respectively, as function of the core/shell ratio. Reference measurements at 300 K show zero coercivity and no EB effect.

For all of the shown parameters (superparamagnetic blocking temperature, coercivity, and EB field), an increase is observed from the as-prepared sample to the first sample of the oxidation series. After this first increase, there is a clearly observable decrease for all parameters. We assume that this behavior is due to the conversion of the magnetite shell that is formed during the synthesis [27] to the maghemite phase in the initial oxidation while the shell is also growing. However, the magnetization measurements for the as-prepared and oxidized samples show no evidence of the Verwey transition expected at around 120 K for the magnetite phase [33]. However, it was observed that the Verwey transition is shifted to much lower temperatures or even vanishes for particles smaller than 6 nm [34]. The shell thickness of the investigated particles is even below this value. Additionally, it should be noted, that the observation of the Verwey transition requires an exact stoichiometry of the magnetite phase. Another reason for the slightly different behavior of the first sample of the oxidation series could be the re-dispersion of the as-prepared sample in fresh 1-octadecene, allowing the oleic acid that covers the particles surface to be re-dissolved and form a new chemical equilibrium. The surface coating in general has an influence on the magnetic properties [35–38]. However, we assume that in our experiments the coating has only a minor influence because of two reasons: (i) The average value of the gaps among the particles of the oxidation series shown in Figure 3, measured from TEM images, ranges between 1.4 and 1.8 nm (with a standard deviation of about 0.4 nm) for the different samples without a visible trend correlating with the oxidation time. Therefore, the coating remains there and particles stay dispersed. (ii) The oxidation is completed at around 250 °C, well before reaching the maximum temperature of 318 °C used for the synthesis. Since the coating is formed at and does withstand the maximum temperature during the initial synthesis, we assume that there is no substantial change at the significantly lower temperatures used during the controlled oxidation process.

The decrease of the superparamagnetic blocking temperature is unexpected. During the oxidation, the volume of the ferrimagnetic spinel phase is increasing; hence, since *T*_B_ = *K*_eff_V/25*k*_B_ [19], an increase of the blocking temperature would be expected. Here, *K*_eff_ is the magnetic anisotropy constant, *V* is the volume, and *k*_B_ is the Boltzmann constant. However, it seems that the antiferromagnetic core biases the shell and influences *K*_eff_. During the oxidation process, the core/shell ratio changes; hence, *K*_eff_ seems to decrease. This explanation is only possible for *T < T*_Neel_, where *T*_Neel_ is the Néel temperature of the AFM core, which ranges for bulk wustite between 192 and 202 K [16,39], close to the superparamagentic blocking temperature of samples S2_0−3_. Other studies found nanosized wustite to behave ferrimagnetically [40] because of spinel-type defect clusters in the wustite lattice [41]. From our measurements, we cannot give a final explanation for the decrease of the superparamagentic blocking temperature for decreasing core/shell ratio. The coercivity shows a similar trend at 10 K. It increases first slightly for sample S2_1_ before it drops down and converges around 25 mT for the fully oxidized particles. This matches with literature values that are ranging from 17 up to 40 mT for maghemite nanoparticles at low temperatures [42–43]. Also, the EB field shows this trend. The core–shell interface leads to a significant biasing effect. At temperatures below 200 K, the core is expected to be antiferromagnetic [16,39]. This coincides with the observed superparamagnetic blocking temperature, which is below 200 K for all samples except sample S2_1_. The resulting EB field measured at 10 K decreases with decreasing shell size from initially about 50 to 1.3 mT. As for *T*_B_ and *H*_C_, sample S2_1_ shows also a maximum of about 56 mT for the EB field at 10 K. The following decrease is expected because of the vanishing EB effect towards core-free maghemite particles caused by (i) a reduction of the interface area and (ii) a reduced volume of the AFM core that leads to a reduced anisotropy energy of the core, which is easier to overcome during the FiM reversal [44]. The residual EB field of about 1.3 mT might be attributed to the surface since this is a source of spin-disorder and frustration [45–46]. This value matches quite well with values reported in the work of Lak and coworkers [15]. There, for 13 nm iron oxide particles, no EB field was found, while larger (core–shell) particles of 18 and 24 nm show values of about 100 and 400 mT, respectively, at 5 K.

#### Superspin glass state

The FC curves (like in Figure 5b) show a slight decrease of the magnetic moment below the superparamagnetic blocking temperature. The ZFC curve shows a significant decrease of the magnetization below *T*_B_. This can be an indicator for a spin glass system [47], which occurs typically at low temperatures and reduces the moment of the sample. However, the FC warming and cooling curves show no thermal hysteresis contrary to the observation of Chandra et al. [48] for such systems. Furthermore, superparamagnetic particle ensembles can also exhibit a superspin glass state characterized by a disordered arrangement of “superspins” below a specific glass transition temperature. This state arises from competing magnetic interactions and geometrical frustrations, leading to a frozen-in magnetic state with collective, disordered behavior. It is often observed in systems with both superparamagnetic nanoparticles and interactions between them, such as those mediated by an antiferromagnetic matrix or nanoparticles with interparticle interactions as in our case. This results in so called “memory effects” that can be observed by intermittent FC experiments [49–50].

**Figure 7:**
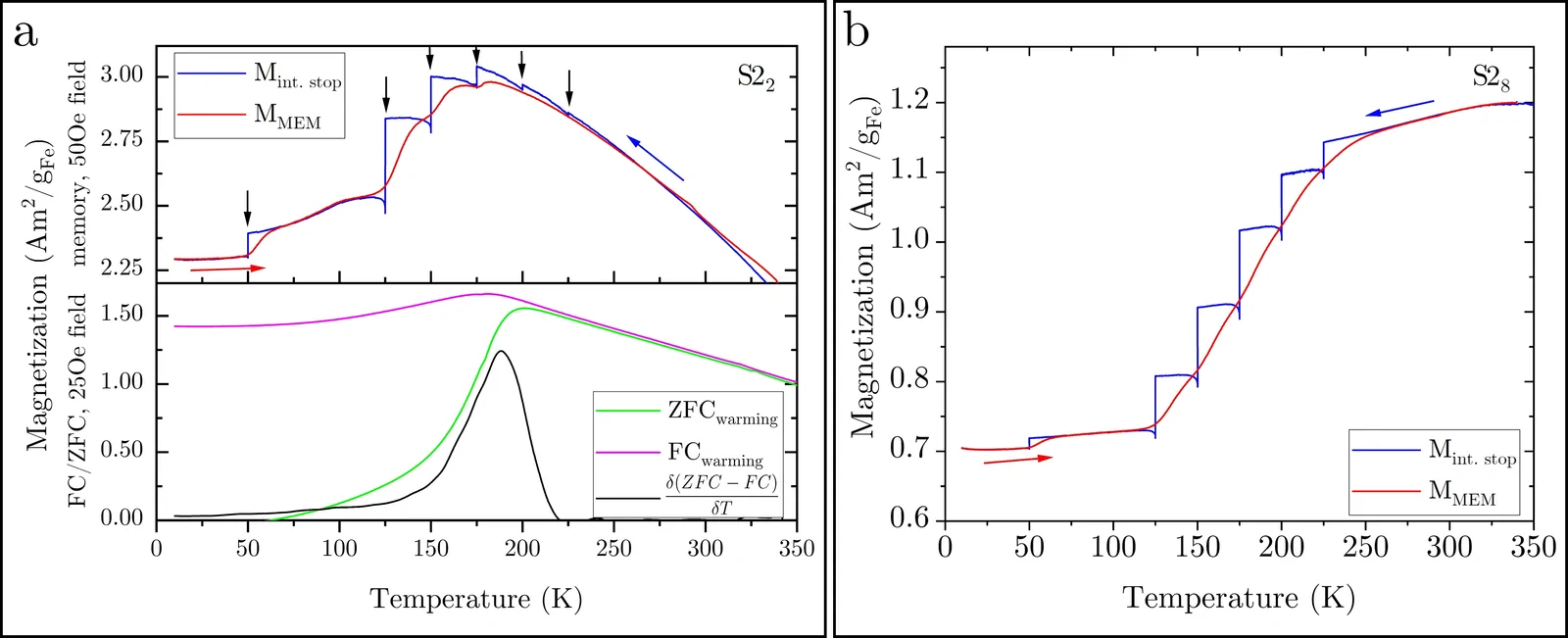
(a) Upper diagram: memory effect (FC). The black arrows mark the break positions during cooling (blue curve, blue arrow). The subsequent *M* vs *T* measurement during warming shows a clear memory behavior (steps in the red curve; measured with a guiding field of 50 Oe). Lower diagram: FC/ZFC curves for comparison (measured with a guiding field of 25 Oe). (b) Memory effect for the oxidized sample S2_8_.

To shed more light onto this behavior, memory experiments were performed, as shown in Figure 7a for sample S2_2_ together with FC/ZFC measurements. The upper diagram shows the memory measurement (red and blue curve), and the lower diagram shows the FC/ZFC measurement (magenta and green curve). As mentioned above, during the cooling process in the FC experiment (blue curve, *M*_int.stop_), several intermittent stops were made, as indicated by the black arrows, followed by the subsequent warming curve showing clearly a memory effect (*M*_MEM_). At every stop, the guiding field of 50 Oe was switched off and the temperature was kept constant for 10^4^ s, before the cooling was continued under 50 Oe. The FC magnetization curve (*M*_int.stop_) during this intermittent-stop experiment shows a drop in the magnetization at every intermittent stop. This arises from the equilibration of the moments. The drop depends on the temperature, being maximal around 125 K. When continuing the cooling, the field of 50 Oe is switched on again, leading to some magnetization recovery. This effect depends on how fast the particles react to the external field. Above *T*_B_, there is almost no relaxation and the recovery is almost 100% since the moments are not blocked. At low temperatures (50 K), the recovery is not observable because of the slow dynamics of the system. In between, there is a significant relaxation observable. After reaching 10 K, a warming curve (*M*_MEM_, red) was measured without stops at a 50 Oe field. This curve clearly contains the well-known memory steps below *T*_B_ [45,49]. A conventional FC/ZFC plot without stops of the same sample is included in Figure 7a (lower diagram) for reference and *T*_B_ estimation. From this, it can clearly be seen that the memory effect vanishes above *T*_B_. Note the matching of the onset of the memory effect with the appearance of the superparamagnetic blocking temperature at 192 K. The different magnetization values are due to different measurement fields of 50 and 25 Oe for the memory and FC/ZFC measurements, respectively. The memory effect is significantly weaker for the fully oxidized sample (S2_8_), as shown in Figure 7b. Here, only at 50 K a memory step is observable in *M*_MEM_. Such memory effects are related with slow dynamics in atomic spin glasses as well as in particle systems with interacting superspins. Besides this, even systems of isolated nanoparticles (without interaction) with a temperature-dependent distribution of relaxation times (originating from the anisotropy energy barriers of the individual nanoparticles) can show memory effects similar to those of spin-glass phases [50–51]. We assumed to have isolated particles due to the dispersion of the particles in the resin matrix first; however, we can not fully exclude the formation of particle clusters with interacting particles and, as can be seen from the results in the following section, there is some interacting of the particles present in the sample.

**Figure 8:**
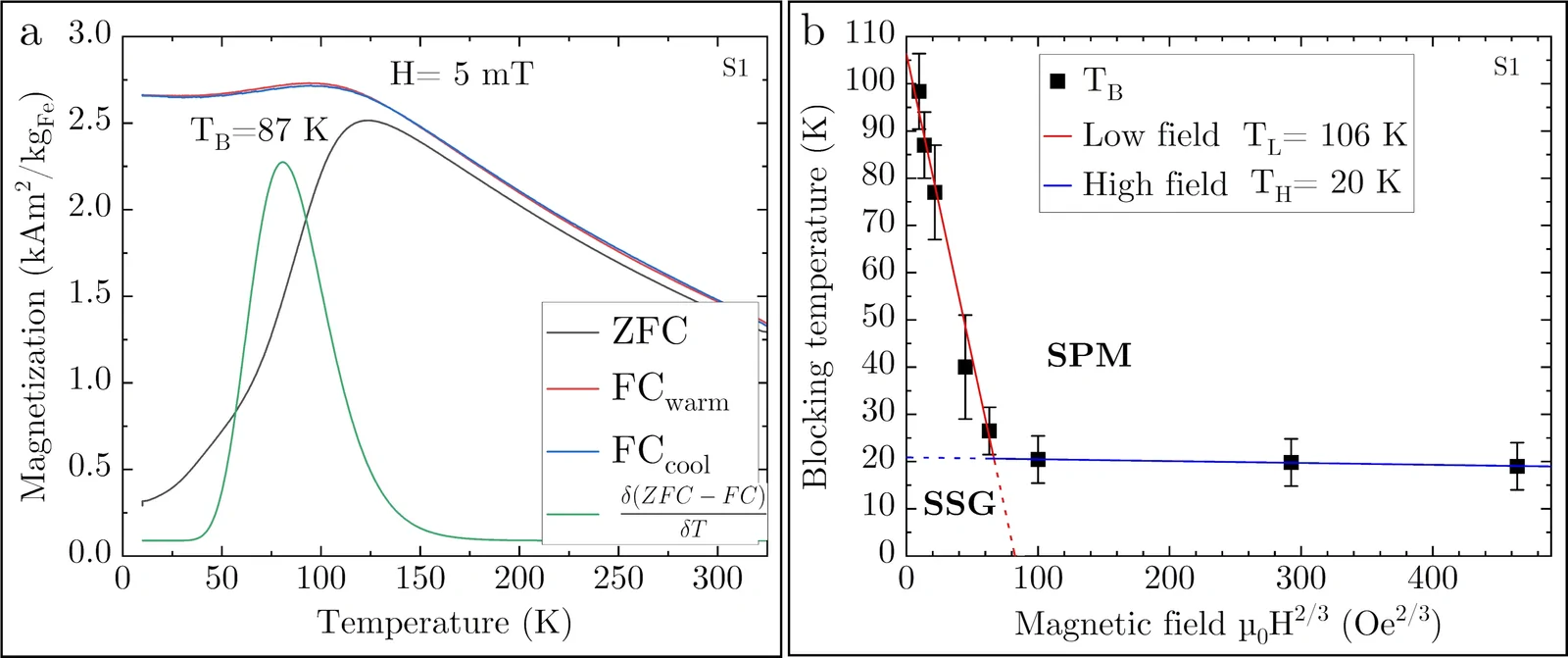
(a) FC/ZFC measurement of sample S1. (b) *T*_B_ vs *H*^2/3^ with Almeida–Thouless lines [48,52] for sample S1. SPM and SSG denote the superparamagnetic and superspin glass region, respectively.

Furthermore, since the blocking behavior depends strongly on the applied field *H*, field-dependent measurements were performed with sample S1. As can be seen from Figure 8a, *T*_B_ of this sample is lower than that of sample S2 due to the smaller particle size, but the temperature-dependent magnetization behavior is qualitatively similar. For superparamagnetic single-domain particles, it is expected that *T*_B_ shifts to lower values for higher guiding fields. The exact behavior is given by the magnetic anisotropy, dipole–dipole-interactions, and fraction of disordered spins [48]. For a critical field, *T*_B_ should vanish. The evolution of *T*_B_ with the applied guiding field *H* is shown in Figure 8b. Here, two regimes can be observed in the *H*–*T*_B_ plane. This suggests the presence of a bimodal energy barrier, which can be assigned to the core and the shell. The blocking temperature follows the Almeida–Thouless law (∼*H*^2/3^) [52] for both regimes, as can be seen from the plot against *H*^2/3^ with linear fits. This is proposed for anisotropic Ising spins, in contrast to the Gabay–Toulouse law [53] for Heisenberg spins, which obeys a *H*^2^ dependence. In our case, a fit with the Almeida–Thouless law gives higher regression values. The lines mark the transition between the glassy and the superparamagnetic behavior [54].

**Figure 9:**
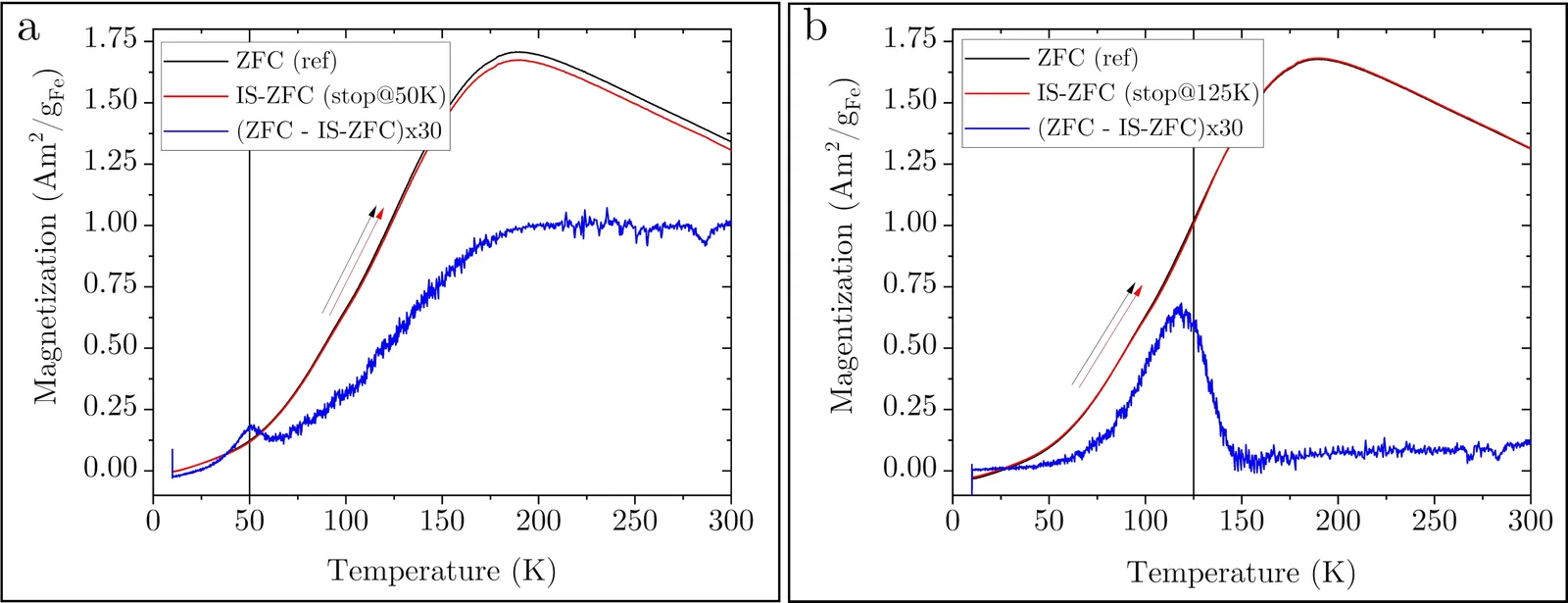
ZFC memory experiment. During ZF cooling, a break of 10^4^ s was made at (a) 50 K and (b) 125 K. The blue curve shows the scaled difference between the warming curve after the intermittent cooling and a reference warming measurement without break. There is a clear peak at (a) 50 K and (b) close to 125 K that indicates a spin glass state.

Besides the FC memory effect, a pure spin glass system shows a memory effect even in the ZFC protocol for an intermittent-stop cooling experiment without applied field. To proof the existence of a superspin glass state, ZFC memory experiments were performed since such systems are expected to show memory behavior even in zero-field experiments, while superparamagnetic particles do not show this effect [49]. Figure 9a presents such an intermittent-stop ZFC experiment with a break at 50 K for 10^4^ s during the zero-field cooling process. The shown curves were both measured during the warming process (indicated by the red and black arrow) after ZFC with and without stop (IS-ZFC and ZFC, respectively). The difference of the curves (multiplied by a factor of 30 to make it visible on the scale) is plotted to reveal the difference between the curves. The peak position in the difference matches perfectly to the break at 50 K. When the break was made at 125 K (Figure 9b), there is a significant shift to 117 K. The reason for this is not clear yet. From this ZFC memory effect, we conclude that the samples show superspin glass behavior; hence, there is significant particle–particle interaction in the samples.

## Conclusion

Cubic monodisperse iron oxide particles with average sizes of 10 and 14 nm, respectively, were produced by thermal decomposition of an iron(III) oleate precursor. The as-prepared particles show a core–shell structure with AFM wustite in the core, surrounded by a FiM spinel shell of γ-Fe_2_O_3_ or Fe_3_O_4_. The particles were artificially oxidized to change the core/shell ratio, until the cores vanished. It was found that the superparamagnetic blocking temperature, the coercivity, and the exchange bias field first slightly increase before they decrease and saturate for the fully oxidized particles without core. Temperature-dependent magnetization measurements show a maximum around the superparamagnetic blocking temperature, and a decreasing magnetization below the blocking temperature is observable for the FC case, which indicates a spin glass state. Furthermore, the particles show memory effects in intermittent FC experiments, which are known for (interacting and non-interacting) superparamagnetic particles. Besides this, even in ZFC experiments a memory effect was observed, which is assigned to the presence of a superspin glass state.

## Data Availability

Data generated and analyzed during this study is available from the corresponding author upon reasonable request.
